# Human Metapneumovirus M2-2 Protein Inhibits Innate Immune Response in Monocyte-Derived Dendritic Cells

**DOI:** 10.1371/journal.pone.0091865

**Published:** 2014-03-11

**Authors:** Junping Ren, Guangliang Liu, Jonathan Go, Deepthi Kolli, Guanping Zhang, Xiaoyong Bao

**Affiliations:** 1 Department of Pediatrics, University of Texas Medical Branch, Galveston, Texas, United States of America; 2 Department of Otorhinolaryngology, Sixth Affiliated Hospital, Sun Yat-Sen University, Guangzhou, China; 3 Institute for Translational Sciences, University of Texas Medical Branch, Galveston, Texas, United States of America; Kantonal Hospital St. Gallen, Switzerland

## Abstract

Human metapneumovirus (hMPV) is a leading cause of lower respiratory infection in young children, the elderly and immunocompromised patients. Repeated hMPV infections occur throughout life. However, immune evasion mechanisms of hMPV infection are largely unknown. Recently, our group has demonstrated that hMPV M2-2 protein, an important virulence factor, contributes to immune evasion in airway epithelial cells by targeting the mitochondrial antiviral-signaling protein (MAVS). Whether M2-2 regulates the innate immunity in human dendritic cells (DC), an important family of immune cells controlling antigen presenting, is currently unknown. We found that human DC infected with a virus lacking M2-2 protein expression (rhMPV-ΔM2-2) produced higher levels of cytokines, chemokines and IFNs, compared to cells infected with wild-type virus (rhMPV-WT), suggesting that M2-2 protein inhibits innate immunity in human DC. In parallel, we found that myeloid differentiation primary response gene 88 (MyD88), an essential adaptor for Toll-like receptors (TLRs), plays a critical role in inducing immune response of human DC, as downregulation of MyD88 by siRNA blocked the induction of immune regulatory molecules by hMPV. Since M2-2 is a cytoplasmic protein, we investigated whether M2-2 interferes with MyD88-mediated antiviral signaling. We found that indeed M2-2 protein associated with MyD88 and inhibited MyD88-dependent gene transcription. In this study, we also identified the domains of M2-2 responsible for its immune inhibitory function in human DC. In summary, our results demonstrate that M2-2 contributes to hMPV immune evasion by inhibiting MyD88-dependent cellular responses in human DC.

## Introduction

Human metapneumovirus (hMPV) is a recently identified human pathogen belonging to the genus *Metapneumovirus* in the *Pneumovirinae* subfamily of the *Paramyxoviridae* family [1]. It is a leading cause of lower respiratory tract disease in children, the elderly and immunocompromised patients worldwide [2]–[5]. hMPV encodes nine proteins. Among them, phosphoprotein P, glycoprotein G, and small hydrophobic SH proteins have been shown to modulate hMPV-induced innate immune response, the first line of host defense against invading pathogens [6]–[9]. Recently, we have identified the M2-2 protein of hMPV is also a major immune suppressor in human airway epithelial cells. M2-2 not only directly targets innate antiviral signaling but also indirectly suppresses anti-hMPV responses by inhibiting the expression of other virulence factors of hMPV, such as G [10]. Whether M2-2 regulates host immunity in other cell types, including human dendritic cells (DC), a family of potent antigen presenting cells (APC), is not currently known.

DC plays a pivotal role in shaping antiviral immune responses in the respiratory tract. DCs can efficiently sense invading pathogens by Toll-like receptors (TLRs) and, because of their strategic localization at mucosal sites, are involved in the response to viral infections [11], [12]. It has been previously shown that hMPV is able to infect human monocytes-derived DC (moDC) and plasmacytoid DC (pDC), and hMPV infection of these two cell-types can effectively block the production of type I IFN in response to TLR agonists [13]. Similarly, following infection with hMPV, mice showed a significant inhibition of IFN-β production in the lung after intranasal inoculation with TLR9 agonist [14]. Since TLRs share common adaptors, such as myeloid differentiation primary response gene 88 (MyD88) and TIR-domain-containing adapter-inducing interferon-β (TRIF), to launch antiviral signaling, hMPV may attack these adaptors, for immune evasion in cells which use TLR to initiate antiviral signaling. We have recently demonstrated that MyD88 is essential for the immune responses of mouse pulmonary conversional DC (cDC) to hMPV infection [15]. Although the regulation of TLR signaling depends on many factors, including species, cell type and TLR in question [16], [17], similar function of MyD88 in hMPV-induced cellular signaling was also identified in human DC. Whether MyD88 is a target of hMPV for immune evasion is not known.

Pattern recognition receptors (PRRs), which include TLRs, DExD/H box RNA helicases, RIG-I and MDA5 (reviewed in [18], [19]), regulate virus-induced innate immune signaling in a cell-type dependent manner. In airway epithelial cells, RIG-I/MAVS-dependent signaling plays a major role in the induction of cytokine, chemokine and type I IFN to control hMPV infection [20]. In monocyte-derived DC (moDC), the activation of antiviral signaling by hMPV requires TLR-4- and MDA5-mediated signaling [21], [22], and TLR-7 is essential for hMPV-induced innate response in pDC [9]. While hosts use various cell-dependent pathways to combat viral infection, viruses also develop immune evasion mechanisms to survive. Regarding hMPV, we have shown it independently uses its G and M2-2 to target RIG-I and MAVS, respectively, to evade the innate immune response of airway epithelial cells [7], [10]. In addition to the inhibitory role of G in airway epithelial cells, hMPV G protein also significantly interferes with TLR-4-signaling in moDC [21]. In this study, M2-2 protein was identified as another key hMPV protein contributing to immune evasion in human DC, since moDC infected with a recombinant hMPV lacking M2-2 protein expression (rhMPV-ΔM2-2) produced higher levels of pro-inflammatory and antiviral immune mediators than moDC infected with wild-type virus (rhMPV-WT). The inhibitory effect of M2-2 on hMPV-induced innate immunity in DC seemed direct and via targeting MyD88. First, M2-2 deletion did not result in the change in the expression of other hMPV proteins, including G in moDC. Second, M2-2 protein suppressed MyD88-dependent gene transcription, but did not affect the gene transcription induced by MyD88 downstream molecules, suggesting M2-2 targets antiviral signaling at the level of MyD88. And third, M2-2, but not M2-1, interacted with MyD88, indicating that the M2-2-MyD88 interaction was M2-2 specific. In summary, our results reveal a novel function of hMPV M2-2 through its targeting of MyD88 in human DC to suppress immune response.

## Materials and Methods

### Cell Lines and Antibodies

LLC-MK2 cells (ATCC, Manassas, VA) and BSR T7/5 cells (baby hamster kidney cells that constitutively express the T7 RNA polymerase, a gift from Dr. Karl-Klaus Conzelmann, Federal Research Center for Virus Diseases of Animals, Germany) were maintained as described [7], [8], [10], [23]. ThP1 cells (ATCC) were cultured in RPMI-1640 medium (Cat #: 22400; Life technologies, Grand Island, NY) containing 10% (v/v) FBS, 1 mM sodium pyruvate, 0.25% Glucose, 100 IU/ml penicillin and 100 µg/ml streptomycin. Monoclonal antibodies against Lamin b was obtained from Sigma-Aldrich (Sigma, St. Louis, MO). Antibodies against MyD88, HA and V5 were purchased from Santa Cruz (Santa Cruz, Santa Cruz, CA), Roche (Roche, Mannheim, Germany) and Invitrogen (Invitrogen, Carlsbad, CA) respectively. The polyclonal rabbit anti-hMPV was raised against purified hMPV. Primary antibodies against IRF-7 and P65 were from Cell Signaling (Cell Signaling, Danvers, MA). FITC-conjugated goat anti-rabbit antibody was from Zymed (Zymed, San Francisco, CA). Horseradish-coupled secondary antibodies were purchased from Santa Cruz (Santa Cruz).

### Purification of moDCs Cells and Viral Infection

Human moDC were generated from human peripheral blood mononuclear cells (PBMC), as previously described [21]. Briefly, the buffy coat was obtained from the UTMB blood bank (approved by Institutional Review Board with IRB# 92–208) and layered on top of Ficoll-hypaque after dextran sedimentation. The layer of mononuclear cells was collected. CD14^+^ cells were isolated by immunomagnetic selection following manufacturer’s instruction (purity >93%) (Miltenyi, Auburn, CA). These CD14^+^ cells were cultured for 7 days in RPMI 1640 supplemented with 2 mMol/liter L-glutamine, 10% FBS, 50 µM 2-mercaptoethanol, and 1,000 U/I penicillin-streptomycin medium containing GM-CSF (100 ng/ml) and IL-4 (20 ng/ml). IL-4 was purchased from R& D Systems (Minneapolis, MN) and recombinant human GM-CSF from PeproTech (Rocky Hill, NJ). One-third of the medium and 100% of each cytokine were replaced every other day. moDCs were used on the seventh day of culture in all experiments.

hMPV CAN-83 and its derived recombinant viruses were propagated in LLC-MK2 cells as we previously described [7], [8], [10]. Viral titer was determined by immunostaining in LLC-MK2 cells, as previously described [7], [8], [10]. To investigate whether M2-2 regulates the cellular responses of moDCs to hMPV infection, cells were infected with naive hMPV, rhMPV-WT or –ΔM2-2 at an MOI of 2 (unless otherwise specified) in the presence of 1 µg/ml trypsin. Uninfected cells or UV-inactivated hMPV-infected cells were used as negative controls. Cell supernatants were harvested 24 h post infection (p.i) to measure cytokine, chemokine, and type I IFN secretion.

### RNA Interference, qRT-PCR and Cytokine/Chemokine Quantification

MyD88 expression was downregulated using 100 nM of siRNA specifically against MyD88 (Sigma, Houston, TX). Non-targeting siRNA were used as negative control. moDCs with siRNA were then exposed to the electroporation system from Amaxa Biosystem, Gaithersburg, MD, as we previously described [21]. At 48 h post-transfection, moDCs were infected with hMPV and harvested at 24 h post-infection (p.i.) to collect cell supernatants and extract total RNA, using Trizol from Invitrogen. MyD88, as well as IL-8 and type I IFN gene expression levels were determined by quantitative real-time PCR (qRT-PCR) as we previously described [7], [10], [20]. The level of cytokines and chemokines in moDC supernatants after infection was quantified by Multi-Analyte Profiling Human Cytokine/Chemokine Kit (Bio-Rad, Hercules, CA), according to the manufacturer’s instructions. Data were analyzed using the Milliplex Analyst Software from Bio-Rad. The IFN-β was accessed by ELISA (PBL Biomedical Laboratories, Piscataway, NJ). We also measured the extracellular lactose dehydrogenase (LDH) after the siRNA treatment to exclude possible indirect and non-specific target inhibition by siRNA-induced cytotoxicity. The LDH was measured by the LDH Cytotoxicity Assay Kit (Thermo Scientific, Rockford, IL), according to the manufacturer’s instruction.

### Reporter Gene Assays

To investigate the role of M2-2 in mediating MyD88-dependent signaling, logarithmically growing 293 were transfected in triplicate with a luciferase reporter gene plasmid containing type I IFN promoter, a plasmid encoding MyD88 or its downstream adaptors IRAK1 or IRAK4 or their respective control vectors, together with a plasmid encoding M2-2 or control viral protein N or its empty vector using FuGene 6 (Roche). Cells were then lysed at 30 h post transfection to measure luciferase, as previously described [7], [10].

### Coimmunoprecipitation (Co-IP)

Logarithmically growing 293 cells in 6-well plates were cotransfected with 2 µg of vector expressing HA-tagged MyD88 (Addgene, Cambridge MA) and 2 µg of plasmid encoding V5-tagged M2-2 protein. Vectors expressing HA or V5 were used as negative control. Cells were harvested 30 h after transfection and immunoprecipitation was carried out using immunoprecipitation kit from Roche (Cat# 11719386001). In brief, 6×10^6^ cells were lysed using 1.5 ml of lysis buffer. A preclearing step was performed by incubating the sample with 50 µl of the protein A/G-agarose for 3 h at 4°C on a rocking platform. Pre-cleared samples were exposed to 5 µg of antibody against either V5 or HA for 1 h at 4°C. An isotype antibody was also included as a control. 50 µl of the protein A/G-agarose were then added to the samples and incubated overnight at 4°C. Immunecomplexes were recovered by centrifugation and washed three times using buffers with different ion strength, provided by the kit. The complexes from the beads were subjected to SDS-PAGE, followed by a Western blot to determine whether MyD88 interacts with M2-2.

We also investigated the interaction between endogenous MyD88 and M2-2 in the context of hMPV infection. To do that, THP1 cells in T75 flasks (10^7^ cells/flask) were mock infected or infected with rhMPV, WT or ΔM2-2, at an MOI of 5 for 24 h. Cells were lysed and immunoprecipitated using an anti-MyD88 antibody or isotype control antibody, as described above. The presence of M2-2 protein in the complex was then detected using an anti-hMPV antibody.

### Western Blot Analysis

The cytosol and nuclear extracts of uninfected and infected cells were prepared using hypotonic/nonionic detergent lysis as described, according to Schaffner protocol [24]. The lysates were collected and quantified with a protein quantification kit from Bio-Rad. Nuclear extracts were fractionated by SDS-PAGE, and transferred to polyvinylidene difluoride (PVDF) membranes. Membranes were blocked with 5% milk in TBS-Tween and incubated with primary antibodies according to manufacturer’s instruction.

### Statistical Analysis

Statistical significance was analyzed using analysis of variance (ANOVA). *P* value of less than 0.05 was considered significant. Mean ± standard error (SE) is shown.

## Results

### MyD88 is Important for hMPV-induced Cellular Response of Human moDCs

TLRs are a class of protein sensors that detect pathogen-associated molecular patterns (PAMPs). Therefore, they play a key role in the innate immune system. Among the 10 members of human TLRs, TLR2, 3, 4, 7, and 8 are involved in the innate immune response to RNA viruses [25]–[27]. On recognition, all TLRs, except TLR3, activates NF-κB, IRF-7 and MAPK through MyD88/IRAK4/IRAK1 pathway, while TLR3 and 4 use their common adaptor TRIF to activate IRF-3 through the TBK-1/IKKε pathway. Among the TLRs, TLR4 is the only receptor that uses both MyD88 and TRIF for cellular signaling [28]. We have recently shown that TLR4 is essential in regulating hMPV-induced cellular signaling in human moDCs, while TLR2 and 3 are not important [21]. In this study, we first investigated whether there is prioritization of MyD88 and TRIF in hMPV-induced cellular signaling DCs.

**Figure 1:**
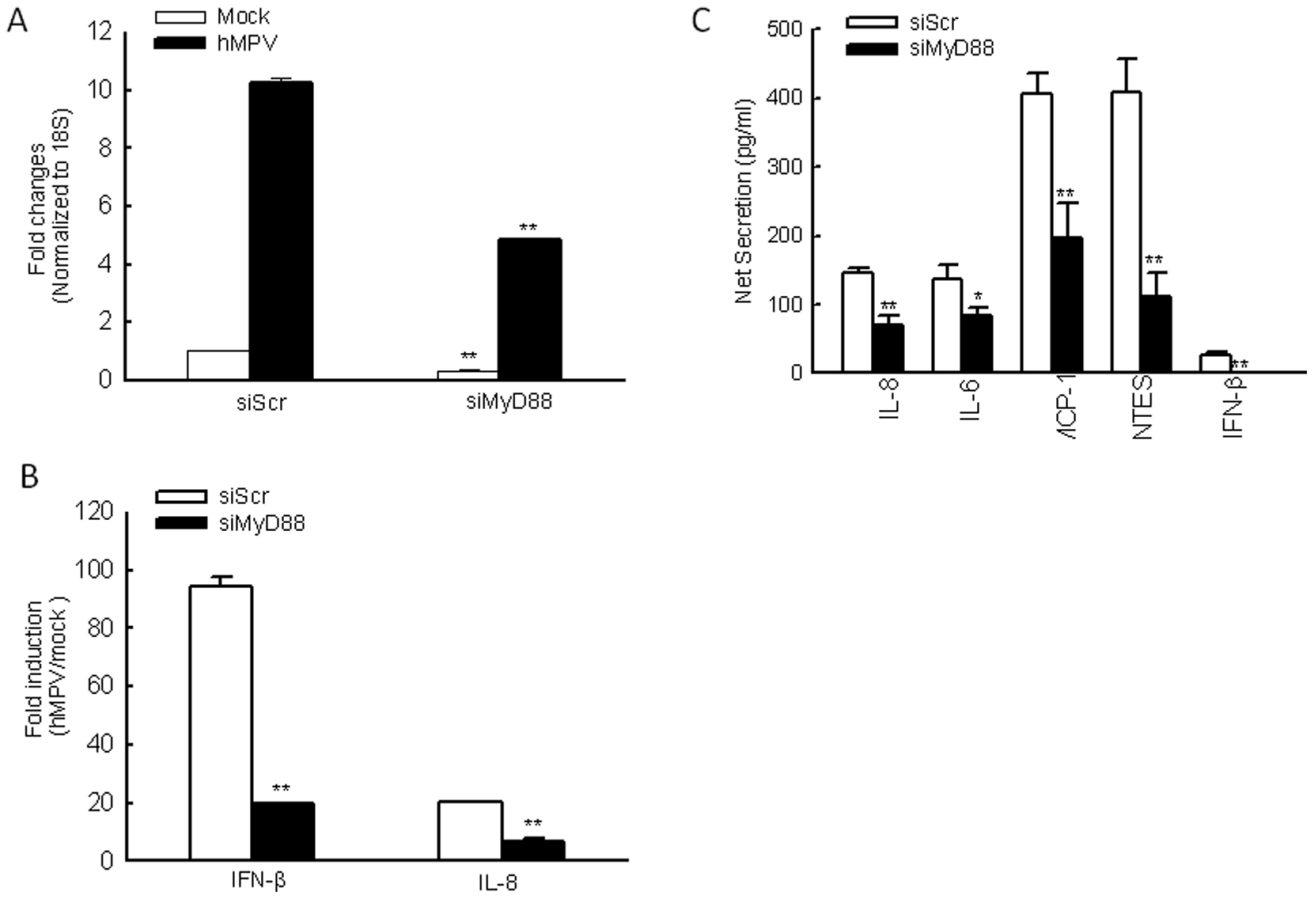
Role of MyD88 in the expression hMPV-induced gene expression. (**A–C**) moDCs were transfected with 100 nM siRNA targeting MyD88 (siMyD88) or a scramble control (SiScr) for 48 h and infected with hMPV at MOI of 5. Cells were harvested at 24 h p.i. to prepare total RNA for analysis of MyD88, IL-8 and IFN-β gene expression by qRT-PCR (**A–B**) and to collect cell supernatants for measuring cytokines, chemokines, and IFN-β secretion by Bio-Plex or ELISA (**C**). Results are representative of two separate experiments. *, *P*<0.05 and **, *P*<0.01 relative to scramble control.

To determine the role of MyD88 and TRIF in hMPV infection, we downregulated their expression in human moDCs, and measured the induction of immune mediators such as type I IFN following hMPV infection. Cells were transfected with either a scrambled siRNA, as control, or one specifically targeting MyD88 or TRIF at a final concentration of 100 nM. Using this concentration, MyD88- or TRIF-specific siRNA inhibited gene expression by about 80%, without affecting cell survival (data not shown). At 48 h post-transfection, moDCs were infected with naive hMPV at MOI of 5 and harvested at 24 h p.i.to collect cell supernatants and extract total RNA. Our results showed that hMPV infection of moDCs significantly enhanced MyD88 expression. The treatment with MyD88 specific siRNA effectively blocked both basal and viral-regulated gene expression (**Fig. 1A**). The basal target downregulation was unlikely due to siRNA-induced cytotoxicity, as the LDH released from cells with scrambled or target-specific siRNA treatment was comparable (**Fig. S1**). There was a significant reduction of IL-8 and IFN-β gene expression in response to hMPV infection in MyD88-silenced cells, compared to scramble siRNA-treated cells **(** **Fig. 1B** **)**. In agreement with our gene expression results, MyD88 silencing resulted in reduced secretion of IL-8, IL-6, MCP-1, RANTES, and IFN-β by hMPV (**Fig. 1C**), demonstrating that MyD88 plays an essential role in hMPV-induced secretion of immune regulatory molecules. However, there was no significant difference in cytokine and chemokine induction, except in RANTES, in control and TRIF-silenced cells, demonstrating that TRIF does not play a major role in host immunity in moDC (**Fig. S2. 1A and B**). In summary, MyD88 played a broader and significant role in regulating hMPV-induced cellular responses in human moDCs.

### hMPV M2-2 Protein Inhibits Viral-induced Cytokine/Chemokine Production in moDC

**Figure 2:**
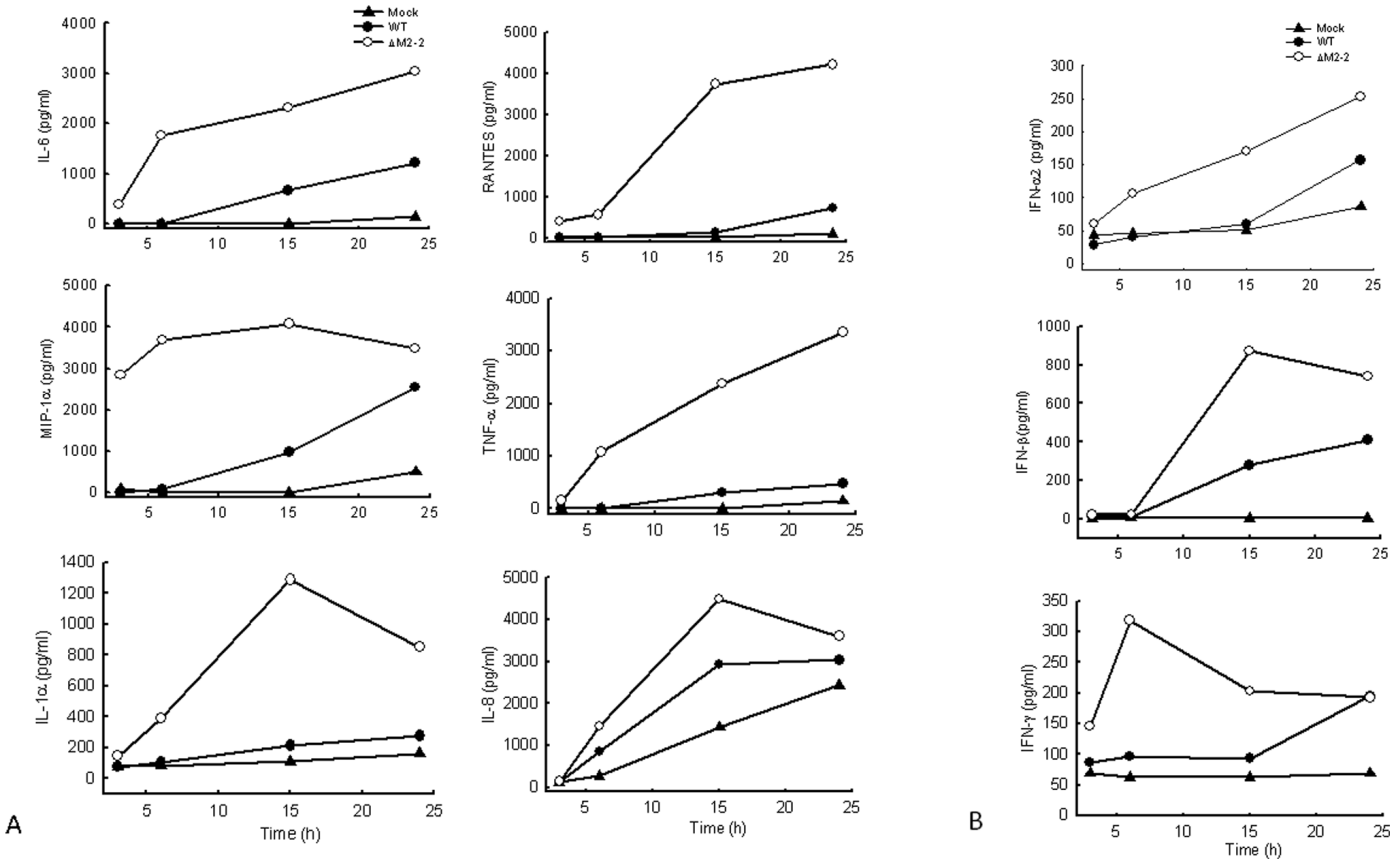
Effect of M2-2 protein deletion on cytokine, chemokine, and IFN secretion. moDCs were infected with hMPV, either WT or ΔM-2, and harvested at different time points p.i. to measure secretion of cytokines, chemokines, (**A**), as well as IFN (**B**), by Bio-Plex or ELISA. Results shown are representative of three separate experiments.

We have recently shown that hMPV M2-2 protein is an important virulence factor, as its expression not only promotes viral RNA synthesis but also directly inhibits viral-induced cytokines, chemokines, as well as IFN production in airway epithelial cells [10] To determine whether this protein plays a role in hMPV-induced signaling in primary immune cells, we infected human moDC with recombinant hMPV, either rhMPV-WT or rhMPV-ΔM2-2, and compared the induction of immune mediator in cell supernatants at various times p.i. moDC infected with rhMPV-ΔM2-2 secreted significantly higher levels of IL-6, IL-8, TNF-α, RANTES, MIP-1α, IL-1α, IFN-α, IFN-β, and IFN-γ, compared to cells infected with rhMPV-WT, at all time points tested (**Figs. 2A and B**), suggesting that M2-2 is a key virulent factor that inhibits hMPV-induced innate immunity in moDC.

**Figure 3:**
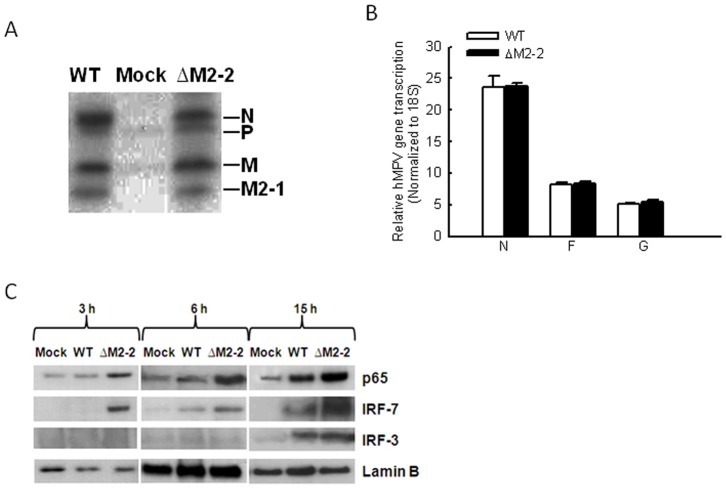
Characterization of WT and ΔM2-2 in moDC. moDC were infected with WT or ΔM2-2 at MOI of 2. Mock infected was used as a control. At 24 h p.i., cells were harvested to prepare total cell lysates or total cell RNA or nuclear extracts. Viral protein expression in total cell lysates was determined by Western blot assay (**A**), viral gene transcription in total RNA samples was assayed by qRT-PCR (**B**), and the nuclear translocation of p64, IRF-7 and IRF-3 was determined by Western blot (**C**). Data are representative of two-three independent experiments.

Since we have previously shown that M2-2 promotes viral RNA synthesis in airway epithelial cells, it is possible that M2-2 also affects viral RNA synthesis in moDC, leading to the changes in other viral protein expression, for example, the previously described virulent fact G protein, and subsequently indirect suppression of host immunity [7], [10]. However, we found that WT- or ΔM2-2-infected moDCs had comparable expression of viral proteins (**Fig. 3**), suggesting the role of M2-2 in viral RNA synthesis is cell-type dependent [13]. As shown in **Fig. 3A**, Western blot using anti-hMPV antibody showed that WT- or ΔM2-2-infected cells have similar expression of viral proteins at 24 h p.i. Consistent with the result shown in **Fig. 3A** **,** qRT-PCR showed that the gene transcription of WT and ΔM2-2 was similar (**Fig. 3B**). The above-described results suggest that M2-2 directly mediates cytokine and chemokine production.

hMPV-induced cytokine and chemokine expression is regulated by transcription factors, including those belonging to NF-κB and IRF family. It has been reported that MyD88 regulates TLR-4/7/8-induced immune responses by activating NF-κB, and is involved in CpG-activated TLR-9 signaling in plasmacytoid DC (pDC) by activating IRF-7 [29]–[32]. To investigate whether M2-2 inhibits the induction of cytokines and chemokines by regulating transcription factors, moDC were infected with WT or ΔM2-2 at MOI of 2. At various time points p.i., cells were harvested for nuclear extract preparation, followed by Western blot analysis to compare the abundance of transcription factors in the nucleus. We found that no p65 and IRF-7 migrated into the nucleus of WT-infected moDC at 3 h p.i., while there was significant concentration of these two transcriptional factors in the nuclear extracts in ΔM2-2-infected moDC (**Fig. 3C**). At 6 h p.i., p65 and IRF-7 started to migrate into the nucleus of WT-infected moDC, and M2-2 deletion further caused more p65 and IRF-7 to migrate into the nucleus. Similar effect of M2-2 in the activation of p65 and IRF-7 was observed in moDC at 15 h p.i. However, M2-2 deletion did not affect nuclear translocation of IRF-3, indicating that M2-2 did not affect hMPV-activated IRF-3. Of note, we did not detect any nuclear translocation of IRF-3 in WT-infected cells at 3 and 6 h p.i., suggesting that the IRF-7 activation occurred earlier than IRF-3 activation and the inhibitory effect of M2-2 on IRF-7 activation at 3 and 6 h pi was likely direct. Since there is no significant induction of type I IFN at 3 and 6 p.i., IRF-7 activation at these two time points was not likely via type I IFN-signaling, as previously described [33].

### M2-2 Targets MyD88

**Figure 4:**
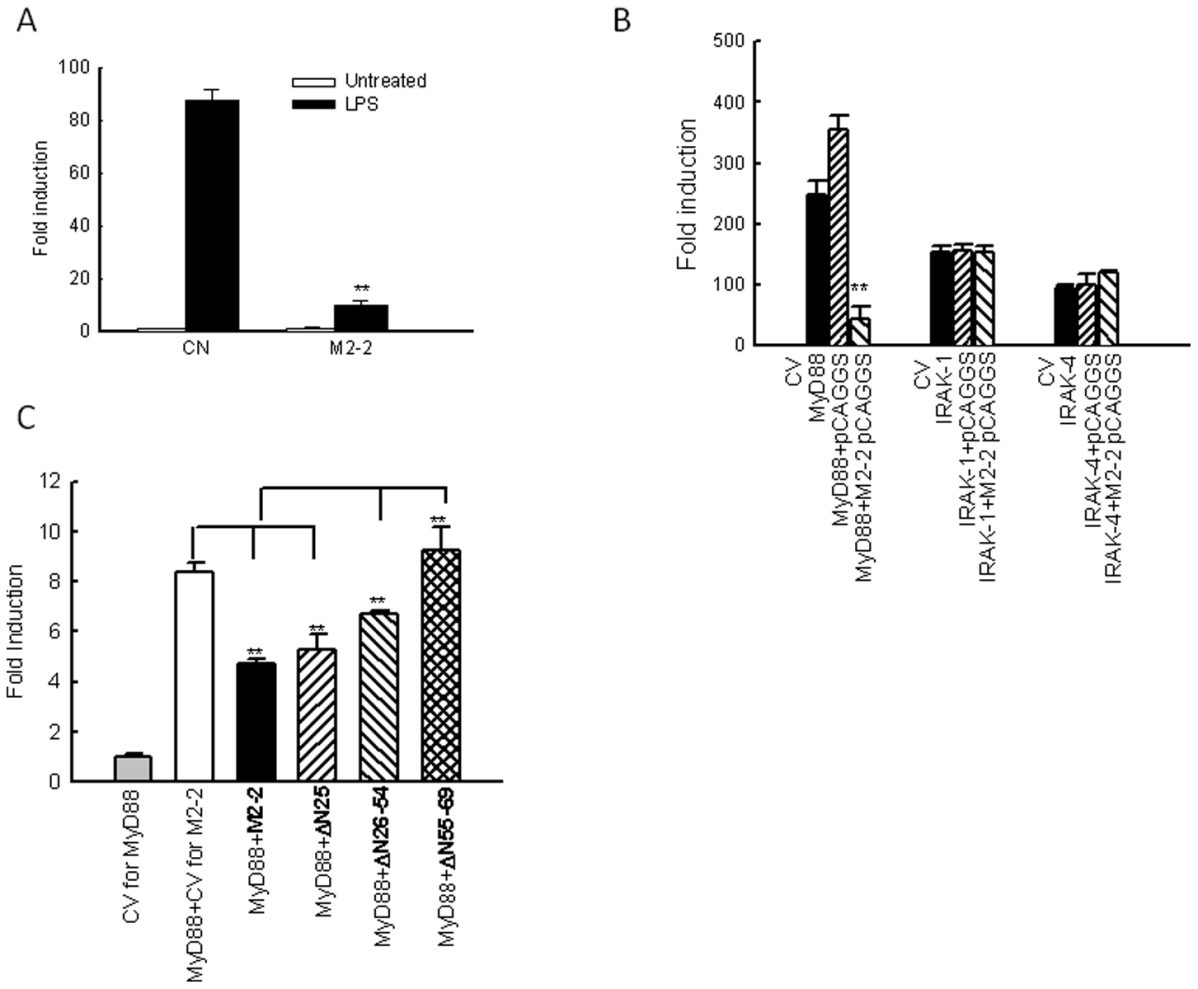
M2-2 inhibits MyD88-mediated signaling. (**A**) M2-2 in suppresses TLR-4 signaling. 293 cells constitutively expressing TLR-4 in triplicates were transfected with a luciferase reporter plasmid under the control of NF-κB binding site NF-κB-Luc (0.05 µg/well), a plasmid encoding M2-2 (0.5 µg/ml) or its control vectors. At 36 h post transfection, cells were either untreated or treated with agonist LPS (100 ng/ml). At 15 h post treatment, cells were harvested for luciferase acitivity measurement. For each plate, luciferase was normalized to the β-galactosidase reporter activity. Data are expressed as means ± SE from 2 experiments. **, *P*<0.01, relative to CN+LPS. (**B**) Inhibition of MyD88-induced IFN-β transcription by M2-2. 293 cells in triplicates (24-well plate) were transfected with a luciferase reporter plasmid (IFN-β-Luc), plasmids encoding either MyD88 (0.2 µg/ml) or their control vectors (CV), or a plasmid expressing hMPV M2-2 or control proteins or the empty vector (0.2 µg/well). Cells were harvested 30 h posttransfection to measure luciferase activity. (**C**) M2-2 domains responsible for MyD88-mediated signaling. 293 cells in triplicates were transfected with IFN-β-Luc (0.2 µg/well), a plasmid encoding MyD88 (0.1 µg/ml) or its control vectors, and a plasmid expressing WT M2-2 or indicated M2-2 mutants or the empty vector. Cells were harvested 30 h posttransfection to measure luciferase activity. For each plate, luciferase was normalized to the β-galactosidase reporter activity. Data are expressed as means ± SE. In panel B **, *P*<0.01, relative to MyD88+ pCAGGS. In Panel C, **, *P*<0.01, column 3 and 4 relative to column 2, and column 5 and 6 relative to column 3.

To elucidate how M2-2 interfered with the cellular response of moDC, we first investigated whether M2-2 inhibits TLR4-mediated signaling, as TLR4-signaling is important for hMPV-induced immune responses in moDC [21]. 293 cells with stable expression of TLR4 (a gift from Dr. Antonella Casola from University of Texas Medical Branch) were transfected with a plasmid containing the luciferase reporter gene under the control of the IFN-β promoter (IFN-β-Luc), and a plasmid encoding M2-2 or its control. After 30 h, the cells were treated with LPS (Escherichia coli K12 LPS; InvivoGen, San Diego, CA) at a final concentration of 100 ng/ml in fresh media, and harvested for measuring the luciferase expression. We found that M2-2 significantly inhibited TLR4 -mediated antiviral signaling (**Fig. 4A**). Interestingly, M2-2 also inhibited TLR7- and TLR9-mediated signaling, as gene transcription induced by TLR7- and TLR-9 ligands, respectively, was significantly suppressed by M2-2 in TLR7- and TLR9-expressing 293 cells (**Fig. S3**). Since TLR4, TLR7, and TLR9 shared a common adaptor, MyD88, and M2-2 is a cytoplasmic protein, it is likely that M2-2 targets MyD88.

To investigate whether MyD88-mediated signaling is inhibited by M2-2, 293 cells were transfected with MyD88 or its downstream signaling molecules IRAK-1/4 expression plasmids, an IFN-β-Luc plasmid, and a plasmid encoding M2-2 or its control. The expression of MyD88 significantly induced IFN-β transcription, which was inhibited by M2-2 protein expression in the absence of viral infection (**Fig. 4B**), suggesting a role of M2-2 in MyD88-mediated signaling. However, IFN-β gene transcription induced by individual expression of IRAK-1 and IRAK-4 was not affected by M2-2, demonstrating that M2-2 blocks DC’s antiviral signaling by targeting MyD88.

We have previously shown that the N-terminus of M2-2 is essential for controlling hMPV gene transcription, while M2-2 regions expanding from Ile26 to Tyr69 are important for inhibiting MAVS-mediated signaling, since M2-2 lacking Ile26- Tyr54 (Δ26–54) and Asn55 to Tyr69 (Δ55–69) significantly impairs the inhibitory role of M2-2, because these two regions contain three and two putative PDZ domains, respectively. (Using motif prediction software online http://elm.eu.org) [34]. We also used these three M2-2 mutants to: 1) confirm the specific role of M2-2 in inhibiting MyD88-mediated antiviral signaling and 2) identify the domains contributing to such inhibition. As illustrated in **Fig. 4C**, the first mutant lacking the first 25 amino acids ΔN25, which does not have putative PDZ domains, had similar inhibitory role with WT M2-2 in blocking MyD88-induced IFN-β transcription, suggesting that this deletion did not affect the inhibitory function of M2-2. However, compared to the inhibitory role of WT M2-2, Δ26–54 and Δ55–69, had diminished ability to inhibit MyD88-induced gene transcription, suggesting that these two domains, possibly PDZ domains, are involved in the signaling regulation, with domain spanning from Asn55 to Tyr69 playing a more robust role in inhibiting MyD88-mediated signaling.

### M2-2 forms a Complex with MyD88

**Figure 5:**
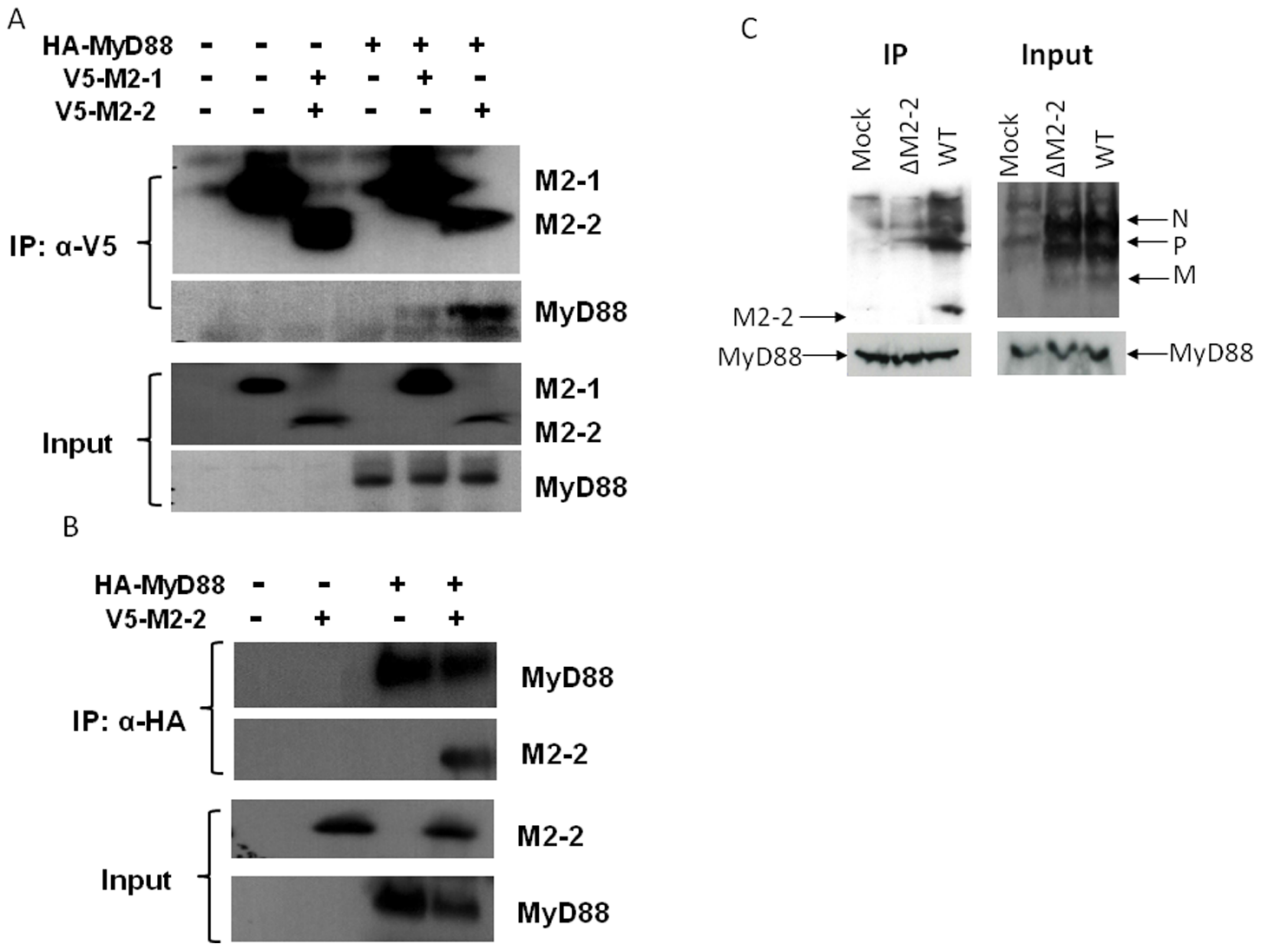
M2-2 interacts with MyD88. (**A–B**) M2-2 forms a complex with MyD88 in the overexpression system. 293 cells were transfected with plasmids encoding HA-tagged MyD88 and V5-tagged M2-2 or their control vectors as indicated. Total cell lysates were immunoprecipitated with an anti-V5 antibody followed by Western blotting using an anti-Flag antibody to detect MyD88 (**A**). Reverse immunoprecipitation was also done, where MyD88 was immunoprecipitated using an anti-HA antibody and M2-2 protein was then detected using an anti-V5 antibody (**B**). Membranes were stripped and reprobed to check for proper IP of M2-2 and MyD88. A small aliquot was also prepared before the IP for a Western blot for equal input of MyD88 and proper expression of M2-2 in 293 cells. (**C**) Viral M2-2 binds to endogenous MyD88 in the context of hMPV infection. THP1 cells were mock infected or infected with rhMPV-WT or -ΔM2-2, at an MOI of 5, and harvested at 24 h p.i. to prepare total cell lysates. Samples were subjected to immunoprecipitation using an anti-MyD88 antibody or control isotype. The immunoprecipitated complexes were then subjected to SDS-PAGE followed by Western blotting using an anti-hMPV antibody. The membrane was then stripped and reprobed with an anti-MyD88 antibody to determine levels of immunoprecipitated MyD88. Data are representative of two independent experiments.

To confirm if MyD88 is a target of M2-2, we investigated whether the M2-2 protein physically interacts with MyD88. 293 cells were transfected with V5-tagged M2-2 and HA-tagged MyD88 expression plasmids. Vectors expressing V5 or HA only were used as negative controls. After 30 h of transfection, cells were lysed followed by immunoprecipitation using anti-V5 antibody (**Fig. 5A**). The immunoprecipitated complex was separated on SDS-PAGE and transferred onto a PVDF membrane. Western blot using anti-HA antibody revealed that MyD88 co-precipitated with M2-2 protein. Reverse immunoprecipitation, using anti-HA to precipitate expressed MyD88 and then using anti-V5 antibody for Western blot, also confirmed that M2-2 was present in the immunoprecipitated complex (**Fig. 5B**). To confirm the specificity of M2-2 in associating with MyD88, we also included hMPV M2-1 protein, a soluble protein with slightly higher molecular weight, into the IP experiments. We found that M2-1 did not bind to MyD88.

The interaction between the M2-2 protein and MyD88 was also investigated in the context of WT and ΔM2-2 infection. THP1 cells, a human monocytic cell line, were used for the infection because the amount of moDC prepared from human peripheral blood mononuclear cells was too limited for the immunoprecipitation. In brief, THP1 cells were mock infected or infected with WT and ΔM2-2 at an MOI of 5, were harvested after 24 h p.i. Total cell lysates were subjected to immunoprecipitation using anti-MyD88 antibodies. The immunoprecipitated complex was separated by SDS-PAGE and transferred onto a PVDF membrane. Western blotting using an anti-hMPV antibody revealed that MyD88 coprecipitated with a protein, which is present only in WT-infected samples and corresponds to the size of identified M2-2 in WT virus particles (**Fig. 5C**), demonstrating that M2-2 associated with endogenous MyD88 in the context of hMPV infection. As discussed in our previous publication, the anti-hMPV antibody is able to detect M2-2 protein of purified hMPV particles. However, it does not detect M2-2 from WT-infected cell lysate, possibly due to its low expression level [10]. IP-enriched M2-2 detected by anti-MyD88 antibody likely met the detection threshold of this anti-hMPV antibody.

## Discussion

As mentioned, hMPV is a major cause of epidemic respiratory infections in infants, the elderly and immunocompromised patients. As a respiratory virus causing repeated infection throughout life, exploring the mechanisms by which hMPV interferes with host antiviral signaling leading to incomplete immune response is urgently needed. Myeloid DC (mDC) in humans are a major stimulator of T cells in paramyxovirus infection [35]. Human moDC represent an appropriate model for lung mDC because monocytes give rise to mDC in the resting lung [36] and mucosa [37], and are phenotypically similar to DC located at the sites of inflammation *in vivo* [38]. In addition, compared to mDC, human moDC are much more readily available and contain similar signaling complexes, both TLR-dependent and independent, than those in mDC. Most importantly, human moDC maintain their regulatory function on T cell activation in response to infection by RSV, a close family member of hMPV, contributing to RSV pathogenesis [39]. In this study, we used moDC as a model of APC to dissect the importance of signaling molecule(s) in the innate immune responses to hMPV infection.

We have previously shown that TLR4 regulates the immune responses of moDC to hMPV infection [21]. Since TLR4 has two adaptors, MyD88 and TRIF, to activate different transcription factors [40], it is important to know whether these two adaptors are equally important for hMPV-induced TLR4-mediated signaling in moDC. We found that MyD88, but not TRIF, played a major role (**Figs. 1** **and S2**), similar to what we have reported in the innate immune responses to hMPV infection in mouse lung cDC [15]. Interestingly, MyD88 was targeted by hMPV M2-2 protein (**Figs. 4** **and** **5**), which may contribute to the suppressed cellular response of moDC to hMPV infection (**Fig. 2**). Since MyD88 is a major adaptor of TLRs (except TLR3), and many immune cells, such as macrophages and pDC, employ TLRs for antiviral signaling [27], [41], [42], it is reasonable to conclude that M2-2 has a common role in suppressing immune responses in other immune cells.

In contrast to the importance of TLR4/MyD88 in mediating hMPV-induced secretion of cytokines and chemokines in moDC, we and others have shown that TLR2, TLR3, and RIG-I do not play such a role in moDC [21], [22]. TLR7 and TLR8, which function as PRRs for RNA viruses, are expressed in moDC [27], [43]. Whether these two TLRs are essential for hMPV-induced innate signaling in moDC is currently unknown. In this study, we found that downregulation of MyD88 suppressed hMPV-induced IL-8 and IFN-β transcription by 75% (**Fig. 1B**), while our previous study showed that TLR4 silencing resulted in lower suppression of these two chemokines (about 50%). Taken together, these results suggest other TLRs in moDC may also function as PRR(s) for hMPV infection. Therefore, downregulation of TLRs’ common adaptor MyD88 had a more profound impact on impairing cellular responses to hMPV than silencing TLR4 alone.

We also showed that M2-2 deletion led to the induction of pro-inflammatory and antiviral mediators by hMPV (**Fig. 2**). By investigating the nuclear translocation of regulatory transcription factors, we found that M2-2 inhibited the activation of p65 and IRF-7, while M2-2 was not involved in IRF-3 activation. IRF-7 is a MyD88-interacting protein and gets activated by TLR9 agonist or DNA virus infection [30], [32], [42]. In addition to MyD88-activated IRF-7, Banos-Lara et.al., has recently demonstrated that MDA5/IRF-3 pathway also played a role in IRF-7 expression in hMPV-infected mice bone marrow-derived DC (BMDC) [22]. We think that M2-2 likely suppressed IRF-7 activation by targeting MyD88, at least at the early time points p.i., but not by regulating IRF-3 activation for three reasons: First, IRF-7 activation by hMPV occurred earlier than IRF-3 activation. Second, IRF-3′s nuclear translocation was not affected by M2-2 (**Fig. 3**). Third, M2-2 interacted with MyD88 (**Fig. 5**). Enhanced IRF-7 activation by M2-2 deletion at early time p.i. was also consistent to increased induction of IFN-α by M2-2 deletion (**Fig. 2B**). Since TRIF did not play a major role in hMPV-induced cytokine/chemokine secretion (**Fig. S2**), MyD88/TRIF pathway might play a minimal role in IRF-3 activation at 15 h p.i. Although MDA5-dependent pathway is critical for IRF-3 activation by hMPV in BMDC [22], and we have previously shown that MAVS, a downstream adaptor of MDA5, is a target of M2-2 in airway epithelial cells [10], we did not observe the change in IRF-3 activation by M2-2 (**Fig. 3C**). Unaffected IRF-3 activation by M2-2 deletion in moDC suggested that MDA5-MAVS may not be important for IRF-3 activation in human DC, or the targeting effect of M2-2 on MAVS is cell type-dependent. Since IRF-7 is an IFN-inducible protein [33], greater IRF-7 expression and consequent enhanced nuclear translocation of IRF-7 at a late time point p.i. might also result from IFN signaling pathway, which was likely to be activated by IFN induction at early time points p.i. Therefore, M2-2-inhibited IRF-7 activation at 15 h p.i. might result from suppressed MyD88/IRF-7 and/or IFN signaling pathways.

Overall, our data demonstrate the importance of MyD88 pathway in regulating innate immune response in hMPV-infected human DC, and the role of M2-2 in mediating MyD88 pathway. These results reveal the nature of virus-host cell interactions that will provide critical insight for the development of novel therapies against hMPV infection, such as a M2-2-based live attenuated vaccine. The role of M2-2 in controlling innate and adaptive immune response to hMPV will be investigated in a future study.

## Supporting Information

Figure S1 **The cytotoxicity of siRNAs.** MoDCs were transfected with a scramble control (SiScr, 100 nM), target-specific siRNA against MyD88 (siMyD88) or TRIF (siTRIF), or actinomycin D (a positive cytotoxic agent, 100 ng/ml) for 48 h. The supernatants were harvested for LDH measurement using the Thermo Scientific LDH Cytotoxicity Assay Kit. Results are from two separate experiments.
(TIF)

Figure S2 **Role of TRIF in the expression hMPV-induced gene expression.** MoDCs were transfected with 100 nM siRNA targeting TRIF (siTRIF) or a scramble control (SiScr) for 48 h and infected with hMPV at MOI of 5. Cells were harvested at 24 h p.i. to prepare total RNA for analysis of TRIF by qRT-PCR (**A**) and to collect cell supernatants for measuring cytokines, chemokines, and IFN-β secretion by Bio-Plex or ELISA (**B**). Results are from two separate experiments. **, *P*<0.01 relative to scramble control.
(TIF)

Figure S3 **Role of M2-2 in suppressing TLR-7/9 signaling.** 293 cells constitutively expressing TLR-7 (**A**) or TLR-9 (**B**) in triplicates were transfected with a luciferase reporter plasmid under the control of NF-κB binding site NF-κB-Luc (0.05 µg/well), a plasmid encoding M2-2 (0.5 µg/ml) or its control vectors. At 36 h post transfection, cells were either untreated or treated with agnonist loxoribine (1 µM) or CpG 2006 (1 nM) for the activation of TLR-7 or TLR-9 respectively. At 15 h post treatment, cells were harvested for luciferase acitivity measurement. For each plate, luciferase was normalized to the β-galactosidase reporter activity. Data are expressed as means ± SE from 2–3 experiments. *, *P*<0.05 and **, *P*<0.01, relative to CN+agonist treated.
(TIF)
